# Comment on “Expanding horizons in burn care: a new paradigm for General Surgeons in Brazil”

**DOI:** 10.1590/0100-6991e-20243823-en

**Published:** 2024-12-02

**Authors:** JOSÉ EDUARDO LUTAIF DOLCI

**Affiliations:** 1 - Associação Médica Brasileira, Diretor Científico - São Paulo - SP - Brasil

Concept of area of expertise:

The area of activity is defined as the modality of organization of medical work, developed by professionals trained to perform specific medical actions, being derived from, and related to, one or more specialties.

Initially, it is essential to highlight the importance and clarity of the article “Broadening horizons in burn care: a new paradigm for general surgeons in Brazil”^1^.

It is a fact that there is a need to considerably expand the training of professionals in “Burn Care”, which is already an area of activity, according to the latest resolution of the Federal Council of Medicine No. 2380/2024 of 06/24/2024, edition 119, section 1, page 145.

This resolution establishes that the training time for this area of expertise is one year, and the necessary requirement is to have a Medical Residency in Plastic Surgery (CNRM) or Title of Specialist in Plastic Surgery (AMB).

I believe that the great and necessary change that must occur to increase the number of “trained” and “legalized” professionals in this area of activity is to increase the specialties involved as necessary prerequisites for obtaining the Title in Burn Care.

Due to the multidisciplinarity involved in this context, I understand that General Surgery, as well as Dermatology, Intensive Care, and Internal Medicine should be able, together with Plastic Surgery, to be a prerequisite for the area of activity “Burn Care”.

I also believe that the optional year of Medical Residency in General Surgery for the training of “Burn Care” makes perfect sense, but it should be a prerequisite for obtaining the title in the area of “Burn Care”, which already exists and does not need to be created again.

Whether the degree will be authorized without the need for a test is a decision that should be made with all the Specialty Societies involved in the area of expertise, which in my view should be at least those mentioned above.

To conclude, it is important to point out that the Brazilian Medical Association supports and praises the spectacular work that is being developed to improve and expand burn care.
